# Effect of 5-year continuous positive airway pressure treatment on MMPs and TIMPs: implications for OSA comorbidities

**DOI:** 10.1038/s41598-020-65029-6

**Published:** 2020-05-25

**Authors:** Beatrix Simon, Imre Barta, Bettina Gabor, Csilla Paska, Gyorgy Boszormenyi Nagy, Eva Vizi, Balazs Antus

**Affiliations:** 1 Department of Pathophysiology, National Koranyi Institute of Pulmonology, Piheno ut 1, H-1121 Budapest, Hungary; 2 Department of Sleep Medicine, National Koranyi Institute of Pulmonology, Piheno ut 1, H-1121 Budapest, Hungary

**Keywords:** Biomarkers, Cardiology, Medical research, Pathogenesis

## Abstract

Continuous positive airway pressure (CPAP) treatment results in nearly complete remission of symptoms of obstructive sleep apnoea (OSA); however, its effect on OSA comorbidities including cardiovascular diseases remains contradictory. Here we investigated the short- and long-term effect of CPAP treatment on matrix metalloproteinases (MMPs) and tissue inhibitors of metalloproteinases (TIMPs) in patients with severe OSA. Serum levels of 7 MMPs and 3 TIMPs were followed in OSA patients (n = 28) with an apnoea-hypopnoea index of ≥30 events/h at the time of diagnosis and at control visits (2 months, 6 months and 5 years) after initiation of fixed-pressure CPAP treatment. The first few months of CPAP therapy resulted in significant decrease of MMP-8 and MMP-9 levels (MMP-8: 146 (79–237) vs. 287 (170–560) pg/mL; MMP-9: 10.1 (7.1–14.1) vs. 12.7 (10.4–15.6) ng/mL, p < 0.05 for each at 2 months), while the rest of the panel remained unchanged as compared to baseline values. In contrast, at 5 years, despite of uninterrupted CPAP treatment and excellent adherence the levels of MMP-8, MMP-9 and TIMPs significantly increased (p < 0.05). Our data suggest that initiation of CPAP therapy leads to a decrease in the level of key MMPs in the short-term; however, this effect is not sustained over the long-term.

## Introduction

Obstructive sleep apnoea (OSA) is characterized by repetitive, complete or partial collapse of the upper airways causing chronic intermittent hypoxia (CIH) and sleep fragmentation. Accumulating evidence suggests that the repetitive sequences of desaturation-reoxygenation lead to increased oxidative stress, sympathetic hyperreactivity, hypertension, progressive endothelial dysfunction, systemic inflammation, hormonal alterations, dyslipidemia and insulin resistance contributing to increased cardiovascular morbidity and mortality in patients with OSA^1,2^.

In recent years a number of new markers have emerged with the potential to predict patient’s risk for cardiovascular disease (CVD) including members of the family of matrix metalloproteinases (MMPs)^3,4^. MMPs are substrate specific endopeptidases that catalyze the degradation of various structural proteins of the extracellular matrix. In health, MMP activity is closely controlled by their specific antagonists, the tissue inhibitors of metalloproteinases (TIMPs). An imbalance of MMPs and TIMPs has been widely implicated in the development of atherosclerosis and its complications^5–8^.

Several lines of evidence indicate that MMPs are important mediators in the process of accelerated atherosclerosis in the CIH-induced oxidative stress in OSA, as well^9^. Accordingly, an early study by Ye *et al*. reported that intermittent hypoxia/reoxygenation was a predictor of enhanced circulating MMP-9 in OSA patients^10^, while Chuang *et al.* found that MMP-9, but not MMP-1, -2, -3 and TIMP-1 increases during sleep in patients with OSA^11^. The contribution of MMP-9 to the development of CVD in OSA has been suggested in other studies as well^12,13^.

Continuous positive airway pressure (CPAP) treatment results in a nearly complete remission of symptoms of OSA. However, the effects of CPAP on OSA comorbidities including cardiovascular outcomes are much less unambiguous. A number of studies have been published on the short-term effects of CPAP on established CVD risk factors, for example on oxidative stress^14^. Regarding MMPs, it was found that 1-month CPAP treatment significantly decreases serum levels of MMP-9 but does not affect TIMP-1 levels in a population of patients with mixed severity of OSA^15^.

Nonetheless, the development of OSA-induced CVDs, and in particular atherosclerosis, is a long and progressive process that is modulated by numerous OSA-independent factors such as systemic inflammation, sympathetic activity, obesity, diet and exercise^16^. Thus, it would be a mistake to extrapolate findings on the short-term effects of CPAP treatment on CVD risk factors and assume that they will be sustained over the long-term. Indeed, the utility of CPAP in preventing CVDs in OSA has been questioned by a recent meta-analysis^17^ that generated interesting pro and con arguments in this field^18,19^.

Therefore, to investigate the long-term effects of CPAP therapy on cardiovascular risk factors, we had initiated a longitudinal study with a 5-year follow-up period in a cohort of patients with newly diagnosed severe OSA. Here we report our findings on MMPs and TIMPs.

## Results

### Enrollment, demographics and clinical characteristics

**Figure 1:**
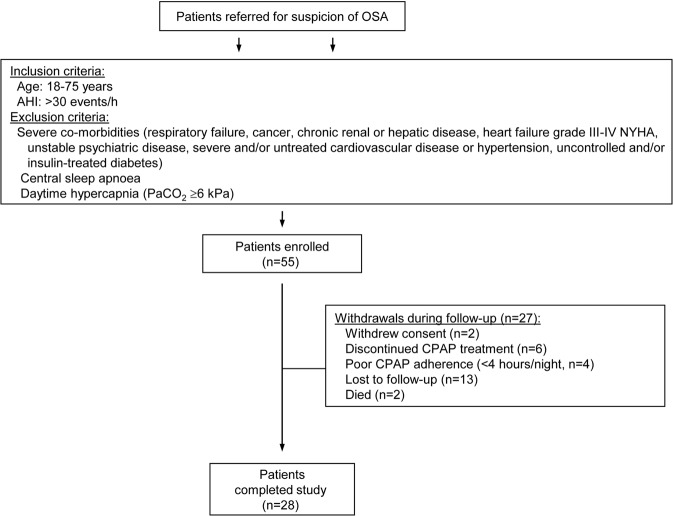
Study flow chart. OSA: obstructive sleep apnoea, CPAP: continuous positive airway pressure, AHI: apnoea-hypopnoea index, PaCO_2_: arterial carbon dioxide tension.

From patients referred to our sleep laboratory for suspicion of OSA during the period of recruitment, 55 fulfilled the criteria for enrollment and agreed to participate (Fig. 1). From these 27 patients had to be withdrawn for various reasons during the follow-up period. Demographic and clinical data of the remaining 28 patients whose serum samples were subjected to MMP array analysis are shown in Table 1.

**Table 1:** Demographic and clinical characteristics of patients who completed the study.

	Measure
*Demographics*	
Subjects (n)	28
Age (years)	54 ± 9.1
Sex (male/female, n, %)	22 (79)/6 (21)
*Smoking history (n, %)*	
Smokers	5 (17.9)
Ex-smokers	9 (32.1)
Non-smokers	14 (50.0)
*Medical history (n, %)*^*#*^	
Hypertension	16 (57.1)
GERD	3 (10.7)
CAD	4 (14.3)
Asthma/COPD	4 (14.3)
Diabetes	2 (7.1)
*Major medications (n, %)*	
Antihypertensives	
Diuretics	3 (10.7)
Ca-channel blockers	4 (14.3)
ACE-inhibitors	3 (10.7)
Beta-blockers	7 (25.0)
Statins	3 (10.7)
Oral antidiabetics	2 (7.1)
Inhaled bronchodilators/corticosteroids	4 (14.3)
Antidiabetics	2 (7.1)
*Pulmonary function*	
FVC (% predicted)	100.7 ± 14.5
FEV_1_ (% predicted)	94.6 ± 20.4
FEV_1_/FVC (%)	74.7 ± 9
*Blood gases*	
PaCO_2_ (kPa)	5.03 ± 0.4
PaO_2_ (kPa)	9.4 ± 1.4

Data are presented as mean ± SD unless stated otherwise. CAD: coronary artery disease, GERD: gastroesophageal reflux disease, COPD: chronic obstructive pulmonary disease, ACE: Angiotensin-converting enzyme, FVC: forced vital capacity, FEV_1_: forced expiratory volume in 1 second, PaCO_2_: arterial carbon dioxide tension, PaO_2_: arterial oxygen tension. ^#^Comorbidities affecting <3% of study subjects were not indicated.

### Effect of CPAP therapy on sleep and clinical variables

Compared to baseline, initiation of CPAP therapy resulted in marked improvements in sleep parameters such as apnoea-hypopnoea index (AHI), oxygen desaturation index (ODI), oxygen saturation SaO_2_), percentage of time in bed (TIB) with <90% oxygen saturation (TIB90%) (p < 0.01 or better for each, Table 2). According to the Epworth sleepiness scale (ESS) score, CPAP therapy normalized subjective sleepiness as well (p < 0.0001). Body mass index (BMI) and C-reactive protein (CRP) levels on the other hand did not change significantly during the 5-year follow up period (p > 0.05).

**Table 2:** Effect of CPAP therapy on clinical and polygraphic variables during follow-up.

	Baseline visit	CPAP		
		2 months	6 months	5 years
*Polygraphic data*				
AHI (events/h)	57.9	1.6	0.6	2.3
	(51.3–72.5 [52–71])	(0.5–2.95 [0.5–2.7])^**^	(0–2.1 [0–2.1])^**^	(1–4.0 [1.6–3.9])^**^
ODI (events/h)	61.1	3	1.8	2.3
	(50–67.5 [53–66])	(2–5.35 [2–4.8])^**^	(0.9–4.5 [0.8–4.6])^**^	(1.1–4.5 [1.4–3.3])^**^
Mean SaO_2_ (%)	90	93	94	94
	(88–94 [90–91])	(92–95 [92–95])^**^	(91–95 [92–94])^*^	(93–95 [94–95])^**^
Minimal SaO_2_ (%)	73	86	85	89
	(65–77 [64–77])	(82–88 [85–88])^**^	(82–89 [76–93])^*^	(86–91 [86–90])^**^
TIB90% (%)	27	0.2	0.1	0
	(14.8–45.0 [20–39])	(0–4.2 [0.1–4])^*^	(0–6.4 [0–2.1])^*^	(0–0.25 [0–0.1])^*^
ESS score	12	3.7	2.9	3.8
	(7.2–14.1 [9–13])	(3.5–5.8 [3–6])^*^	(2.9–4.2 [2–5])^*^	(1.8–7.1 [2–7])^**^
*Laboratory data*				
WBC (×10^9^/L)	7.3 ± 1.5 (6.7–7.9)	6.5 ± 1.3 (6.0–7.1)	7.3 ± 3.2 5.8–8.9)	7.0 ± 1.2 (6.6–7.4)
CRP (mg/L)	8.6 ± 6.4 (5.9–11.2)	6.4 ± 7.4 (3.1–9.7)	9.4 ± 7.1 (6.1–12.7)	6.4 ± 4.5 (4.6–8.1)
Glucose (mmol/L)	6.6 ± 2.3 (5.6–7.5)	6.8 ± 2.5 (5.8–7.9)	6.5 ± 1.8 (5.9–7.4)	5.8 ± 1.4 (5.3–6.3)
BMI (kg/m^2^)	35.7 ± 5.7 (33.5–37.9)	35.8 ± 6.0 (33.3–38.3)	35.1 ± 4.9 (30.9–39.2)	35.7 ± 6.1 (33.2–38.2)
CPAP adherence (h/night)^#^	—	6.07 ± 1.0 (5.9–6.5)	6.09 ± 1.1 (6.0–6.7)	6.47 ± 1.2 (6.0–6.9)

Data are presented as mean ± SD (95% CI) or median (IQR [95% CI]). CPAP: continuous positive airway pressure, AHI: apnoea-hypopnoea index, ODI: oxygen desaturation index, SaO_2_: arterial oxygen saturation, TIB90%: percentage of time in bed with arterial oxygen saturation less than 90%, ESS: Epworth sleepiness scale, WBC: white blood cell count, CRP: C-reactive protein, BMI: body mass index. ^#^CPAP adherence data for the 2 month, 6 month and 5 year visits represent CPAP use in the first two months, months 3–6 and the last 6 months before the end of the study, respectively. *p < 0.01 and ^**^p < 0.0001 vs. baseline visit.

The mean nightly duration of CPAP usage was >4 hours in each subject at 2 and 6 months and the 5-year visit (Table 2). Each subject used the CPAP device >4 hours/day in at least 70% of days throughout the study with 89.6 ± 9.2% in the last 6 months preceding the final visit.

### Early effects of CPAP therapy on serum MMP profile

**Figure 2:**
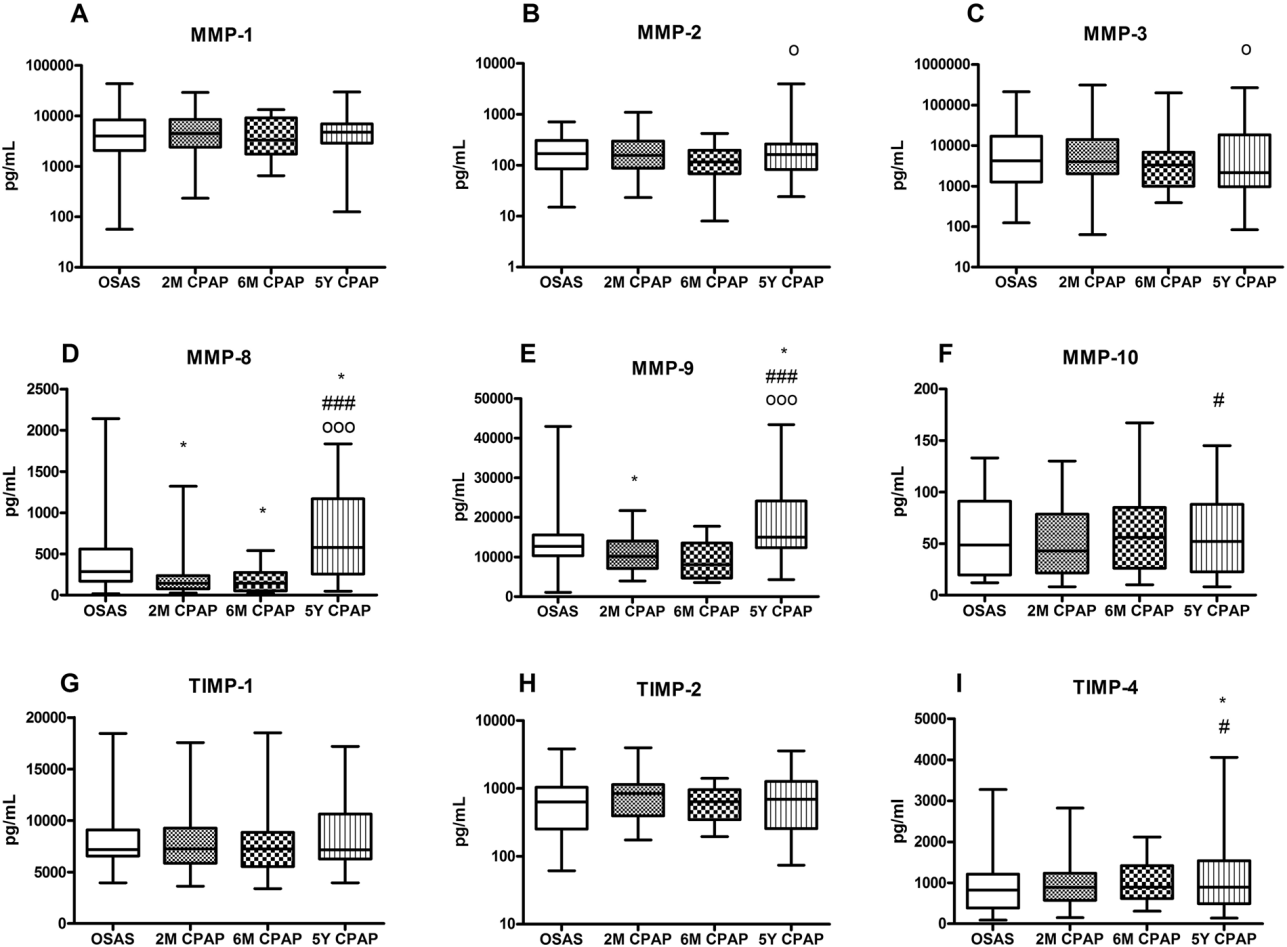
Serum MMP (Panel A–F) and TIMP (Panel G–I) levels of study subjects during the 5-year follow-up. Marker levels were assessed at the time of diagnosis (OSA), at 2 and 6 months (2M and 6M) and at 5 years of CPAP therapy (5Y). OSAS: obstructive sleep apnoea syndrome (baseline visit), CPAP: continuous positive airway pressure, MMP: matrix metalloproteinase, TIMP: tissue inhibitor of matrix metalloproteinase. Standard box plots with median (25^th^ and 75^th^ percentiles) and whiskers (at minimum and maximum values) are shown. *p < 0.05 vs. baseline visit; ^#^p < 0.05 and ^###^p < 0.001 vs. 2-month visit; ^°^p < 0.05 and ^°°°^p < 0.001 vs. 6-month visit.

Using undiluted serum, all analytes but MMP-13 fell within the detection range of the protein array. Serum levels of MMP-8 and MMP-9 markedly decreased at the 2-month control visit as compared to those at the time of OSA diagnosis (146 (79–237 [95% CI 85–217]) vs. 287 (170–560 [95% CI 226–403]) pg/mL at baseline, p = 0.028 and 10.1 (7.1–14.1 [95% CI 7.8–13.2]) vs. 12.7 (10.4–15.6 [95% CI 10.8–15.0]) ng/mL at baseline, p = 0.029, respectively; Fig. 2), while the level of the remaining analytes did not markedly change during this early period of CPAP treatment. At 6 months only MMP-8 was significantly below the level observed at OSA diagnosis, but a similar tendency for a decrease could be observed for MMP-9 as well (146 (54–276 [95% CI 54–276]) vs. 287 (170–560 [95% CI 226–403]) pg/mL at baseline, p = 0.018 and 8.1 (4.7–13.6 [95% CI 4.7–13.6]) vs. 12.7 (10.4–15.6 [95% CI 10.8–15.0]) ng/mL at baseline, p = 0.083, respectively).

### Serum MMP profile after 5 years of CPAP therapy

Despite uninterrupted CPAP therapy, at the 5-year control visit increased levels of MMP-8, MMP-9 and TIMP-4 were detected compared to those at the time of OSA diagnosis (578 (255–1167 [95% CI 295–1070]) vs. 287 (170–560 [95% CI 226–403]) pg/mL at baseline, p = 0.017; 15.0 (12.4–24.2 [95% CI 12.9–22.7]) vs. 12.7 (10.4–15.6 [95% CI 10.8–15.0]) ng/mL at baseline, p = 0.014 and 893 (496–1542 [95% CI 568–1428]) vs. 828 (387–1211 [95% CI 482–1074]) pg/mL at baseline, p = 0.023, respectively; Fig. 2). The 5-year change in the level of other MMPs did not reach statistical significance compared to baseline but some, such as MMP-2 and MMP-10 when compared to early time-points following initiation of CPAP treatment also significantly increased by the end of the study (Fig. 2).

### Subgroup analysis

To explore the possibility that short- and long-term changes in serum MMP-8, MMP-9 and TIMP-4 levels were affected by patient’s characteristics, data were reanalyzed after stratifying patients by median age, BMI and whether or not they have been taking medication for hypertension at the time of OSA diagnosis. However, both at 2 months and at 5 years past CPAP treatment initiation the mean difference in change between these subgroups was similar (Supplementary Table S1).

### Correlations between MMPs and sleep parameters

No correlations were found between MMPs, TIMPs and main polygraphic variables such as AHI, ODI and TIB90% at the time of OSA diagnosis (Supplementary Fig. S1).

### Correlations between MMPs and clinical variables

At baseline, a strong positive association was found between MMP-8 and white blood cell count (WBC) (r = 0.62, p < 0.001; for neutrophils: r = 0.76, p < 0.001) and a weaker one for CRP (r = 0.47, p = 0.019). Notably, both of these correlations disappeared after 2 months of CPAP treatment (r = −0.02, p = 0.933 for WBC; r = −0.04, p = 0.855 for CRP). MMP-3 and MMP-9 showed a weak positive correlation with WBC only at OSA diagnosis (r = 0.40, p = 0.033 and r = 0.43, p = 0.021; respectively). Another potentially important, albeit weak negative correlation was observed between MMP-1 and BMI (r = −0.37, p = 0.049). No other clinically important correlations were detected (Supplementary Fig. S2).

## Discussion

Our study has demonstrated that CPAP therapy in patients with severe OSA results in a marked reduction in the serum levels of MMP-8 and MMP-9 in the short-term, while at the same time it has lesser or no effect on the concentration of several other members of the MMPs and TIMPs. The most striking finding of our study was that the beneficial effect of CPAP on key MMPs implicated in the progression of atherosclerosis was not sustained over the long-term as by the end of the 5 year follow-up period, the levels of MMP-8, MMP-9 and TIMP-4 increased beyond even those detected at the time of OSA diagnosis.

OSA is considered an independent risk factor for CVD. In support of this concept, a very recent study demonstrated marked coronary plaque formation in patients with OSA using coronary computed tomography angiography^20^. As discussed earlier, MMPs have been associated with both oxidative stress and cardiovascular diseases, and hypoxic conditions were shown to influence MMP expression, secretion and activity making these markers potentially important mediators in the process of accelerated atherosclerosis in OSA^10–13,21^.

Notably, it was found that initiation of CPAP treatment of OSA significantly decreases serum levels of MMP-9, but does not affect TIMP-1 levels within 1 month^15^. Our data regarding the short-term effect of CPAP treatment on MMP-9 and TIMP-1 not only reiterates these observations, but also adds new information about the behavior of several other MMPs and TIMPs. Taken together, this is consistent with a scenario where the effects of OSA-induced CIH on CVDs are mediated by only a subset of MMPs, including MMP-8 and MMP-9, and that CPAP therapy alleviates this aspect of CVD risk in the short-term by eliminating the burden of CIH.

Little is known about the long-term effect of OSA on CVD, particularly the impact of CPAP treatment on MMP levels. In our study by the end of the follow-up period, increased levels of MMP-8, MMP-9 and TIMP-4 were measured compared to the time of OSA diagnosis. Our original study design had also called for the inclusion of control subjects i.e. severe OSA patients who for any reason discontinued CPAP therapy during the follow-up period. However, despite our best efforts, for the final visit we were unable to recruit such patients in sufficient numbers required for statistical analysis. Nonetheless, reappearance of CIH can be excluded as a reason behind the elevated MMP-8, MMP-9 and TIMP-4 levels, since the initial strong association of MMP-8 and MMP-9 with markers of systemic inflammation had not reappeared at 5 years, consistent with the high adherence of our patients to CPAP.

Our subgroup analysis was carried out to see if independent CVD risk factors such as age, BMI and a history of hypertension exerted significant influence on the extent of change in the levels of MMP-8, MMP-9 and TIMP-4 during the 5-year follow-up period. Stratification of the subjects based on the above parameters did not uncover differences between the subgroups suggesting that at least in this cohort of patients, age, BMI and hypertension did not play a major role on the short and long term changes of these analytes. It must be noted, however, that every single member in our cohort of patients was obese at the time of OSA diagnosis, and initiation of CPAP therapy did not prompt a change in their lifestyle. Moreover, by the end of the 5-year follow-up period the cohort-wide mean change of BMI was 0.9 kg/m^2^, while the SD of the change was only 2.14, with over 80% of the patients had less than 5% change in their BMI. Obesity as a constant comorbid condition could explain why the initially beneficial effect of CPAP could not be sustained in this cohort.

It was shown that obesity and an associated low-grade systemic inflammation modulates MMP-9 levels in children with OSA, independently from OSA severity^16^. The authors found that BMI and CRP levels correlate with MMP-9 levels and speculated that a more severe CIH may be responsible for the higher prevalence of systemic inflammation in adults with OSA. On the other hand, there is evidence that exercise and diet lowers MMP-9 levels within 2 weeks as was shown in a cohort of overweight children^22^. The expression and activity of MMPs are regulated by various hormones and growth factors, including insulin, leptin and adiponectin, factors involved in adipose tissue expansion^23^. It is therefore conceivable that the lack of exercise and diet that led to invariable BMI in our cohort of severe OSA patients may be responsible for the increased levels of a number of MMPs by perpetuating a low grade systemic inflammation, independent of OSA.

We detected positive correlations of MMP-8 with neutrophils and CRP and between MMP-9 and neutrophils at the time of OSA diagnosis. Associations between MMPs and markers of systemic inflammation are not unprecedented; increased serum levels of CRP and MMP-9 in OSA had been reported before^11^. The fact that all these correlations disappeared after only 2 months of CPAP treatment is another strong indication that MMP-8 and MMP-9 are central in mediating the systemic effects of CIH.

In conclusion, in this study we investigated the effect of CPAP therapy, both short- and long-term, on the MMP profile of patients with severe OSA. We observed that CPAP therapy in the short-term lowers the serum concentration of MMPs formerly implicated as CVD risk factors, but these potentially beneficial effects were not sustained over the long term. CPAP treatment may only eliminate some but not all MMP associated cardiovascular risk in obese patients with severe OSA.

## Methods

### Study patients

Subjects newly diagnosed with severe OSA (AHI ≥30 events/h) at the sleep laboratory of our Institute were recruited to participate in the study during a period of 18 months starting in 2011. Inclusion and exclusion criteria are detailed in Fig. 1. The research protocol was approved by the National Ethics Committee (OGYÉI/29037). All procedures performed in the study involving human participants were in accordance with the 1964 Helsinki declaration and its later amendments or comparable ethical standards. All subjects gave written informed consent to participate in the study.

### Study design

Diagnosis of OSA was established by overnight polygraphy (SOMNOscreen RC, SOMNOmedics GmbH, Electro Oxygen Ltd., Hungary) as described in detail earlier^24^. The AHI, the ODI, the TIB90% and the mean and the lowest SaO_2_ were recorded and used as indexes for OSA severity. In the morning potential participants diagnosed with severe OSA were further evaluated that included assessment of blood gases, lung function, BMI, smoking habit, comorbidities and ESS. Patients fulfilling all inclusion/exclusion criteria were enrolled (baseline visit) and fasting blood samples were collected for routine laboratory tests and for research purposes. Blood gases and lung function were measured as previously described^25^.

In the night following diagnosis optimal pressure for CPAP therapy was titrated. Patients started using fixed-pressure CPAP the day after titration.

At 2 month and again at 6 month after the initiation of CPAP therapy patients were reviewed at the sleep laboratory. At these visits polygraphy was repeated, adherence to CPAP was assessed (when needed, CPAP pressure was adjusted), and in the morning routine blood chemistry was performed.

Patients were advised to attend the sleep laboratory for routine ambulatory check-up at least once in two years. The final visit was scheduled at 5 years after diagnosis at which point the sleep study (using the patients’ own CPAP device) and all other procedures listed above for the baseline visit were repeated.

### CPAP adherence

CPAP adherence was calculated at each scheduled visit from data recorded by the patient’s own CPAP device (S8 or S9 Escape models, ResMed). The percentage of days on which CPAP was used >4 hours per night was documented as well.

### MMP and TIMP measurements

Serum MMP-1, -2, -3, -8, -9, -10, -13 and TIMP-1, -2, -4 levels were measured using antibody arrays (Quantibody Human MMP Array 1; RayBiotech, Norcross, GA, USA) following the manufacturer’s protocol. Slides were scanned by Genepix 4300 A reader (Molecular Devices). Signal intensities on MMP arrays were calculated by subtracting local background from medians of the four parallel signal intensities.

### Statistical analysis

Parametric data are presented as mean ± SD with 95% confidence intervals (CI) of mean while median with interquartile ranges (IQR) and 95% CI of median is used for variables with non-parametric distributions. Data normality was tested by the Kolmogorov-Smirnov test. Changes in MMP and TIMP levels were analyzed pair-wise by the Wilcoxon signed rank test. Clinical and polygraphic variables were analyzed either by the Friedman test followed by the Dunns test (non-parametric data) or repeated-measures analysis of variance with post hoc test (Newman-Keuls test) for multiple comparisons (parametric data). Correlation coefficients were calculated by Spearman’s method. Calculations were performed by GraphPad Prism (GraphPad Software Inc., San Diego, CA, USA). The threshold of significance was set at p < 0.05.

## Supplementary information

Supplementary information.

## Data Availability

The datasets generated and analyzed during this study are available from the corresponding author on reasonable request.
