# Supplementary material for: Effect of 5-year continuous positive airway pressure treatment on MMPs and TIMPs: implications for OSA comorbidities
Source: Sci Rep. 2020 May 25;10:8609. doi: 10.1038/s41598-020-65029-6 (PMC7248085; doi:10.1038/s41598-020-65029-6)
Supplement: Supplementary file 1 — Supplementary information. [file 41598_2020_65029_MOESM1_ESM.pdf]

Supplementary Material

Effect of 5-year continuous positive airway pressure treatment on MMPs and TIMPs: implications for OSA comorbidities

Beatrix Simon[1,2], Imre Barta[1], Bettina Gabor[2], Csilla Paska[1], Gyorgy Boszormenyi Nagy[2], Eva Vizi[2], Balazs Antus[1,2*]

1 Department of Pathophysiology, National Koranyi Institute of Pulmonology, Piheno ut 1, H-1121 Budapest, Hungary

2 Department of Sleep Medicine, National Koranyi Institute of Pulmonology, Piheno ut 1, H-1121 Budapest, Hungary

***Corresponding author:** Balazs Antus, MD, DSc; Department of Pathophysiology, National Koranyi Institute of Pulmonology**,** Piheno ut 1**,** H-1121 Budapest**,** Hungary**;** Telephone: +36 1 391 3309**,** Fax: + 36 1 200 7060**,** Email: antusb@hotmail.com

Supplementary Table S1.

Mean changes in MMP-8 and MMP-9 and TIMP-4 levels after 2 months and 5 years of CPAP treatment in patients stratified by the median age and body mass index and whether or not they have been taking medication for hypertension at the time of OSA diagnosis.

	MMP-8		MMP-9		TIMP-4	
	Δ2M-BL pg/mL	Δ5Y-BL pg/mL	Δ2M-BL pg/mL	Δ5Y-BL pg/mL	Δ2M-BL pg/mL	Δ5Y-BL pg/mL
**Age**						
<56 years old (n=15)	-137 (-366-91)	473 (225-722)	-3057 (-6914-800)	5085 (1019-9152)	-34 (-206-139)	311 (-132-755)
≥56 years old (n=13)	-257 (-674-160)	2 (-429-433)	-4041 (-10652-2570)	2935 (-1639-7510)	28 (-97-153)	239 (-93-571)
**BMI**						
<35 kg/m^2 (n=14)	-304 (-687-79)	195 (-218-609)	-5317 (-11788-1155)	5393 (797-9989)	34 (-98-167)	379 (-157-914)
>35 kg/m^2 (n=14)	-85 (-311-142)	313 (5-622)	-1804 (-5003-1395)	2782 (-1151-6714)	-44 (-220-131)	177 (4-351)
**HT**						
no HT (n=12)	-29 (-332-274)	252 (-177-681)	-1256 (-4444-1932)	3240 (-223-6703)	26 (-151-202	191 (-151-533)
treated for HT (n=16)	-297 (-591-(-)4)	256 (-66-577)	-4979 (-10300-342)	4722 (92-9353)	-28 (-173-117)	343 (-77-763)

Data are presented as mean (95% confidence intervals). OSA: obstructive sleep apnoea, CPAP: continuous positive airway pressure, MMP: matrix metalloproteinase, TIMP: tissue inhibitor of matrix metalloproteinase, 2M: 2 months of CPAP treatment, 5Y: 5 years of CPAP treatment, BL: baseline, HT: hypertension, BMI: body mass index

Supplementary Fig. S1.

Correlations between MMPs/TIMPs and main polygraphic variables and heart rate at OSA diagnosis. P-values of Spearman correlations are indicated in the cell for each pair and gray scale coded according to the label at right. OSA: obstructive sleep apnoea, MMP: matrix metalloproteinase, TIMP: tissue inhibitor of matrix metalloproteinase, ESS: Epworth sleepiness scale, AHI: apnoea-hypopnoea index, ODI: oxygen desaturation index, TIB90%: percentage of time in bed with arterial oxygen saturation less than 90%, HR: heart rate

	ESS score	AHI	ODI	TIB90%	max.HR	min.HR	avg.HR
MMP-1	0.884	0.376	0.276	0.950	0.384	0.964	0.872
MMP-2	0.422	0.224	0.072	0.667	0.760	0.907	0.373
MMP-3	0.513	0.227	0.112	0.745	0.399	0.999	0.505
MMP-8	0.255	0.956	0.967	0.786	0.468	0.771	0.879
MMP-9	0.122	0.200	0.314	0.902	0.728	0.117	0.049
MMP-10	0.378	0.787	0.433	0.694	0.160	0.096	0.002
TIMP-1	0.836	0.301	0.097	0.796	0.841	0.387	0.178
TIMP-2	0.817	0.222	0.342	0.565	0.788	0.848	0.430
TIMP-4	0.501	0.352	0.270	0.343	0.521	0.212	0.044

p<0.001

p<0.01

p<0.05

p>0.05

OSA diagnosis

Supplementary Fig. S2.

Correlations between MMPs/TIMPs and clinical variables at OSA diagnosis (Panel A) and after 5 years of CPAP treatment (Panel B). P-values of Spearman correlations are indicated in the cell for each pair and gray scale coded according to the label at right. OSA: obstructive sleep apnoea, CPAP: continuous positive airway pressure, MMP: matrix metalloproteinase, TIMP: tissue inhibitor of matrix metalloproteinase, HDL-C: high-density lipoprotein cholesterol, FVC: forced vital capacity, FEV_1: forced expiratory volume in 1 second, CRP: C-reactive protein, WBC: white blood cell count, $PaCO_2$: arterial carbon dioxide tension, PaO_2: arterial oxygen tension, BMI: body mass index

A

	age	tricglicerid	total cholesterol	HDL-C	glucose	CRP	WBC	pO2	pCO2	BMI
MMP-1	0.060	0.883	0.863	0.754	0.622	0.156	0.863	0.313	0.337	0.050
MMP-2	0.246	0.942	0.548	0.492	0.675	0.227	0.624	0.521	0.541	0.983
MMP-3	0.160	0.327	0.232	0.969	0.274	0.491	0.033	0.880	0.399	0.871
MMP-8	0.673	0.524	0.301	0.183	0.519	0.019	34e-0	0.291	1.000	0.329
MMP-9	0.437	0.701	0.921	0.525	0.816	0.184	0.021	0.437	0.648	0.931
MMP-10	0.314	0.732	0.798	0.835	0.063	0.345	0.094	0.948	0.816	0.328
TIMP-1	0.375	0.989	0.962	0.313	0.949	0.251	0.533	0.912	0.250	0.391
TIMP-2	0.159	0.560	0.551	0.223	0.101	0.583	0.089	0.708	0.066	0.564
TIMP-4	0.233	0.827	0.805	0.728	0.565	0.962	0.971	0.613	0.108	0.863

OSA diagnosis

B

	age at diag.	tricglicerid	total cholesterol	HDL-C	glucose	CRP	WBC	pO2	pCO2	BMI
MMP-1	0.105	0.507	0.917	0.120	0.597	0.458	0.583	0.786	0.666	0.114
MMP-2	0.963	0.907	0.055	0.745	0.131	0.994	0.538	0.474	0.024	0.656
MMP-3	0.037	0.416	0.862	0.607	0.316	0.423	0.879	0.572	0.438	0.611
MMP-8	0.433	0.883	0.747	0.821	0.787	0.491	0.188	0.873	0.897	0.342
MMP-9	0.865	0.335	0.406	0.313	0.997	0.219	0.259	0.595	0.431	0.149
MMP-10	0.371	0.859	0.379	0.457	0.155	0.540	0.539	0.199	0.638	0.130
TIMP-1	0.444	0.956	0.754	0.149	0.557	0.277	0.527	0.943	0.947	0.260
TIMP-2	0.222	0.200	0.307	0.690	0.977	0.336	0.438	0.397	0.608	0.435
TIMP-4	0.427	0.921	0.890	0.698	0.494	0.008	0.564	0.202	0.534	0.568

5-year CPAP

$p<0.001$

$p<0.01$

$p<0.05$

$p>0.05$
